# Letter to the editor of Heliyon re: A novel compound heterozygous variation in the FKBP10 gene causes Bruck syndrome without congenital contractures: A case report [Heliyon 2024; 10(7): e28680]

**DOI:** 10.1016/j.heliyon.2024.e40336

**Published:** 2024-11-19

**Authors:** Omar Abdulmalek, Khadeja Alrefaie

**Affiliations:** University of Glasgow, United Kingdom; Royal College of Surgeons in Ireland, Bahrain

**Abstract**

To the Editor of Heliyon,

The recent article by Shang et al. [1] details a case involving osteopenia, fragility fractures, lumbar kyphosis, and dentinogenesis imperfecta (DI), but notably absent are congenital joint contractures. Yet, the case is classified as “Bruck syndrome type 1 (BS1) without congenital joint contractures or OI type XI.” This classification hinges on identified genetic mutations in the FKBP10 gene, specifically a novel compound heterozygous variation including mutations c.23dupG and c.825dupC, which lead to frameshift and premature stop codons.

While the genetic analysis is thorough, the designation of this condition as BS1 merits reconsideration. According to the Online Mendelian Inheritance in Man (OMIM) database, BS1 is characterized by congenital contractures with pterygia, frequent fractures in infancy or early childhood, short stature, severe limb deformity, and progressive scoliosis—[2] contrasting starkly with the definition of OI type XI, which involves bone fragility and low bone mass without contractures [3].

The absence of joint contractures in the presented patient does not meet the traditional diagnostic criteria for BS1, which invariably includes such contractures. Therefore, the notion of “Bruck syndrome without congenital contractures” is inconsistent with the established diagnostic criteria. Instead, the symptoms align more closely with OI type XI, sharing the same genetic mutations and similar clinical manifestations but lacking contractures. Additionally, the presence of DI in the patient complicates the classification further. The authors correctly note that DI is rarely seen in BS1. However, this acknowledgment raises a contradiction in their diagnostic approach. By recognizing that DI is more typical of OI type XI, yet still classifying the case as BS1—despite the absence of congenital joint contractures—the authors themselves introduce a significant controversy. This contradictory stance on the symptoms associated with FKBP10 mutations undermines the clarity of their diagnosis and highlights a potential misalignment with established clinical characteristics.

It is misleading to default to a BS1 or OI type XI classification based solely on the FKBP10 gene mutation, without fully considering the clinical presentation. BS1 is a clinical diagnosis defined by both bone fragility and congenital contractures, a critical component missing in this case. Hence, it is inaccurate to label the patient's condition as BS1 when the defining clinical feature is absent.

This diagnostic ambiguity could lead to confusion in clinical management and genetic counseling. We suggest that this case be more accurately classified as osteogenesis imperfecta type XI, which reflects the genetic findings and clinical presentation without the contractures characteristic of BS1.

We urge a reassessment of the phenotypic classification used in this study to prevent potential misdiagnosis and to support precision in the ongoing research into the molecular pathology of these conditions.

Subject: Letter to the editor of Heliyon re: A novel compound heterozygous variation in the FKBP10 gene causes Bruck syndrome without congenital contractures: A case report. Heliyon. 2024; 10(7): e28680.

## CRediT authorship contribution statement

**Omar Abdulmalek:** Conceptualization, Supervision, Writing – original draft, Writing – review & editing. **Khadeja Alrefaie:** Conceptualization, Writing – original draft, Writing – review & editing.

## CRediT authorship contribution statement

**Omar Abdulmalek:** Conceptualization, Supervision, Writing – original draft, Writing – review & editing. **Khadeja Alrefaie:** Conceptualization, Writing – original draft, Writing – review & editing.

## Declaration of competing interest

The authors declare that they have no known competing financial interests or personal relationships that could have appeared to influence the work reported in this paper.
